# Changes in physicochemical parameters of the alpine/mountain stream influenced by summer flash flood in Tatra Mountains (Western Carpathians)

**DOI:** 10.1007/s10661-024-12835-4

**Published:** 2024-06-24

**Authors:** Jaroslav Solár, Tatiana Pitoňáková, Andrea Pogányová

**Affiliations:** Institute of High Mountain Biology, University of Zilina, Tatranská Javorina 7, 059 56 Tatranska Javorina, Slovak Republic

**Keywords:** Alpine/mountain stream, Physicochemical parameter of water, Flash flood, Tatra Mountains

## Abstract

Changes to the physicochemical parameters of water in alpine/mountain streams can provide evidence of ongoing natural and anthropogenic processes in their catchment. In this study, we analysed a mountain stream (Javorinka) on the north-eastern side of the Tatra Mountains (Western Carpathians), which is minimally influenced by human activity. The stream was monitored weekly for 5 years (2017–2021) and evaluated for its seasonal variations in physicochemical parameters. These seasonal variations were influenced by the large summer flash flood in July 2018. We hypothesise that floods are essential for the oligotrophic profile of alpine/mountain streams. To support this idea, our main objective was to compare the seasonal trends of the main physicochemical parameters in the stream before and after floods or periods of high flow. We found evidence to support our hypothesis. For example, there was a significant decrease in the chemical consumption of oxygen and ammonia, and, conversely, an increase in the ratio of saturated oxygen and nitrate concentrations. Stream bed erosion also resulted in increased phosphates (over the next 2 years) and high enrichment of the water by dissolved solids in the spring. Interestingly outside of the main objectives, we observed a significant decrease in sulphates, especially in the summer and autumn of 2020 and 2021, which may be related to suppressed emissions due to the restriction of the COVID-19 lockdown. The observed trends and their changes therefore support the idea that alpine/mountain streams are excellent indicators of ongoing environmental processes, and that occasional summer flash floods support the oligotrophic profile of the stream system.

## Introduction

For ecologists studying freshwater ecosystems, the physicochemical conditions of water in streams and lakes are crucial for understanding how various organisms interact with their environment and each other. These interactions, and their effects, are often deeply rooted in the state of the environment and its periodic or random changes. Alpine/mountain streams, due to their environmental heterogeneity, are habitats with significant diversity, often including endemic species that are well adapted to harsh and cold conditions (Hotaling et al., 2017). However, these ecosystems are fragile due to complex climate-hydrology limiting factors that influence the spatiotemporal heterogeneity of water sources (Michel et al., 2020) and the physicochemical parameters of streams (Robinson et al., 2022). The ‘quality of the aquatic environment’ is reflected by factors mostly influenced by natural cycles or disturbances (droughts, flash floods), as well as anthropogenic processes. Mountains act as barriers to air masses, which are usually rich in precipitation and long-range emissions (Ballová et al., 2020; Janiga & Haas, 2019; Kopáček et al., 2006).

In alpine/mountain environments, the physicochemical conditions of water are strongly related to the type of system (stream, spring, or lake), its feed (rain, snow, glacier), position, altitude, and hydrogeology (Żelazny et al., 2018). These conditions are primarily influenced by meteorological, hydrological, and geological-lithological factors (Sajdak et al., 2018). Seasonal patterns follow hydrological cycles driven by the accumulation of water sources in a catchment. For example, high flow reduces the contribution of weathering products in water, but snowmelt in winter or spring increases the contribution of compounds flushed from the soil (Foks et al., 2018). In winter, streams are oversaturated with weathering products or groundwater (Donnini et al., 2016), while from April to July they are undersaturated (Maher et al., 2009). Hydrochemical processes can be exacerbated by temperature or storm events, resulting in a high phosphorus (Ockenden et al., 2016) or nitrogen loads (Feinson et al., 2016; Fovet et al., 2018). Large storm events or flash (or extreme) floods can significantly increase erosion, alter the stream morphology, and flush large amounts of material (Mazzorana et al., 2018), reducing the abundance of individual organisms in the stream (Janiga et al., 2021; Smith et al., 2019). Various ecological processes support the ecological memory of aquatic ecosystems (Baho et al., 2021; Hughes et al., 2019), with extreme events driving the long-term preservation of these systems in oligotrophic conditions.

Monitoring of alpine/mountain streams is essential because water is a crucial component of any ecosystem, and water from mountain streams is a vital source of freshwater for lowland ecosystems and human communities. Understanding ongoing processes in streams to maintain their quantity and quality will continue to be critically important in the face of climate change and human population growth. This study provides observations collected over 5 years on natural processes related to physicochemical parameters of Javorinka stream, an alpine/mountain stream in the Tatra Mountains (West Carpathians), minimally affected by human activities. During the monitoring period, we recorded an extreme flash flood (2018) and we focused on how this event affected water parameters and their seasonal trends in the stream. We hypothesise that floods are essential for maintaining the oligotrophic profile of alpine/mountain streams. To support this idea, our main objective was to compare the seasonal trends of the main physicochemical parameters in the stream before and after floods or periods of high flow.

## Materials and methods

### Study area

We chose the Javorinka because it is an alpine and mountain stream in the Tatra Mountains (Western Carpathians, Europe) which is minimally influenced by human activity. The stream is fed by snow-rain precipitation and originates in the upper parts of the main mountain ridge of the High Tatras. The stream flows through the Javorová Valley, which has naturally well-developed subnival, alpine, sub-alpine, and mountain zones. The area is strictly protected as part of the National Nature Reserve Javorová Valley in the Tatra National Park (Slovakia), established in 1948. Our sampling site was located above the first signs of settlement in the mountain village of Tatranská Javorina (N 49.2593711°; E 20.1435901°; 1,015 m a.s.l.). At high altitudes, there is only evidence of a forester’s hut and a water supply system for the nearest settlements. Geologically, the stream flows mainly through a granite valley, but 2.8 km above the sampling site there is a right tributary (Meďodolský potok) from a valley of various sedimentary rocks (limestones, dolomites, quartz sandstones, and clay shales). The Tatra Mountains form a barrier to air masses (especially from the northwest), creating a distinctive mountain climate characterised by sudden and often extreme weather changes. At the sampling site, the average temperature in July is 16 °C and total annual precipitation is 1512 mm (Bičárová & Holko, 2013). The stream has a 5° slope, a width of 9 m, and an average water depth of 0.7 m at the sampling site. The average annual discharge over the last 20 years was 1.899m^3^ s^−1^ (data from the nearest station Ždiar-Podspády-Javorinka of the Slovak Hydrometeorological Institute; approximately 4 km below the sampling site). The streambed consists of boulders, gravels, and riparian sands. The surrounding area consisting of partly managed mountain spruce forest, and stands of Norway spruce (*Picea abies*), grey alder (*Alnus incana*), and mixed willow species on the riparian zone.

### Sampling and sample processing

The study site was monitored weekly from January 2017 to the end of December 2021. In situ, we monitored basic water parameters such as water temperature (°C), pH, conductivity (COND in µS/cm), total dissolved solids (TDS in mg/l), dissolved oxygen content (DO in mg/l), oxygen partial pressure (*p*O_2_ in mbar), and dissolved oxygen saturation (DO_%_ in %). We used a portable multimeter WTW 3430 (GEOTECH, Weilheim, Germany) with compatible probes: IDS pH electrode (Sen TixR 940–3), conductivity electrode (TetraCon 925–3), and optical oxygen electrode (FDO 925–3). Measurements were taken 1 m from the stream bank in a flowing section of the stream. Water for laboratory analysis was collected in dark glass sterile bottles (2 l) and stored in a refrigerator. Samples were processed immediately, or up to 12 h after sampling. In the laboratory, we monitored chemical parameters such as nitrates (N, NO_3_ in mg/l), ammonia (N, NH_3_, NH_4_ in mg/l), chlorides (Cl, NaCl in mg/l), sulphates (S, SO_4_^2−^ in mg/l), phosphates (P, PO_4_^3−^ in mg/l), and total hardness (CaCO_3_ in mg/l) using the photometer YSI EcoSense 9500 (YSI Inc., OH, USA) and appropriate chemical reagents (PALINTEST Ltd., Gateshead, UK). Chemical oxygen demand (COD in mg/l) was determined using a standardised manganometric (KMnO_4_) titration method according to Horáková et al. (1989). All measurements (in situ and laboratory) were repeated three times and then averaged with a relative standard deviation of less than 5%.

### Statistical analysis

The complete dataset of measured values was supplemented by daily mean flow data provided by the Slovak Hydrometeorological Institute, which monitors flows in the Javorinka stream approximately 4 km downstream of the study site (Ždiar-Podspády-Javorinka station). Based on the flow records, we sorted the weekly data into three groups: measurements on days with higher flow than 5 m^3^ s^−1^, measurements in the week before high flow, and measurements in the week after high flow. This process was necessarily for evaluation how high flow can affect water parameters in the stream. Statistical analyses were mainly focused to compare pre-flood and post-flood data. The July 2018 flash flood served as a significant event for this comparison. Statistical analysis was performed using Statistica 12 (StatSoft, USA). As the data were not normally distributed (according to the Shapiro–Wilk normality test), non-parametric approaches were used. The Mann–Whitney *U* test (M-W *U*) or a Kruskal–Wallis H test (K-W H) was used to test for differences. Principal component analysis (PCA) based on correlations was performed to better estimate the mutual relationships between the measured variables. Data were normalised by *z*-score standardisation prior to the PCA procedure. The resulting principal component coordinates of cases were tested for seasonal influence (years, seasons, and months) using analysis of variance (ANOVA).

## Results

### Seasonal variations in physicochemical parameters and impact of the 2018 summer flash flood

During the 5-year monitoring period (2017–2021), significant differences were observed between individual years in most variables, with the exception of water temperature, dissolved oxygen content (DO), and CaCO_3_ values. In July 2018, a significant rainfall event (217.9 mm, SHMÚ, 2018) caused a huge flash flood, increasing flow from 2.48 to 25.94 m^3^/s, leading to massive erosion of the stream bed and bank. This flood event likely impacted year-over-year differences in almost all studied variables except flow, temperature, pH, DO, CaCO_3_, and sulphates (Table 1). Consequently, we focused on all periods with a high flow that did not have such a devastating effect.

**Table 1:** Differences of physicochemical variables measured over 5 years (2017–2021) in mountain stream Javorinka in the Tatra Mountains (Slovakia). Differences were computed with regard to seasons and months (values represented *p*-values of K-W H test and significant differences less 0.05 are bolded). Letters indicate significant seasonal/monthly differences in individual years of monitoring (a, 2017; b, 2018; c, 2019; d, 2020; e, 2021). Column ‘Flood’ represents differences between data before and after the flood in July 2018. Column ‘High flows’ represents differences between three groups of data: (1) measurements in days with higher flow than 5 m^3^ s^−1^, (2) measurements the week before high flows, and (3) measurements the week after high flows (values represented *p*-values of M-W *U* test and significant differences less 0.05 are bolded)

Variables	Years	Seasons		Seasons in years		Months	Months in years	Flood before/after	High flows		
									1/2	2/3	1/3
Flow day mean m^3^/s	**0.049**		**0.001**	abcde	**0.001**		abcde	0.437	**0.000**	**0.006**	**0.000**
*t* °C	0.875		**0.001**	abcde	**0.001**		abcde	0.245	0.597	0.795	0.653
COD mg/l	**0.001**		0.768	b de	0.776		ab de	**0.001**	0.189	0.667	0.321
pH	**0.001**		**0.037**	bcde	**0.043**		cde	0.258	**0.010**	0.088	0.258
DO mg/l	0.439		**0.001**	abcde	**0.001**		abcde	0.564	0.209	0.680	0.375
*p*O_2_ mbar	**0.001**		**0.003**	abc e	0.086		b e	**0.001**	0.587	0.700	0.791
DO_%_	**0.001**		**0.004**	abcde	**0.057**		abc e	**0.001**	0.384	0.879	0.511
COND µS/cm	**0.011**		**0.000**	abcde	**0.001**		abcde	**0.003**	**0.003**	0.121	0.099
TDS (mg/l)	**0.010**		**0.000**	abcde	**0.001**		abcde	**0.002**	**0.001**	0.068	0.059
Cl mg/l	**0.001**		**0.047**		0.471		d	**0.001**	0.732	0.408	0.533
NaCl mg/l	**0.001**		0.064		0.566		d	**0.001**	0.658	0.374	0.580
CaCO_3_ mg/l	0.121		**0.001**	abcde	**0.001**		abcde	0.210	0.337	0.914	0.126
SO_4_^2−^ mg/l	**0.001**		**0.001**	abcde	**0.001**		abcde	0.439	0.547	0.993	0.694
S mg/l	**0.001**		**0.001**	abcde	**0.001**		abcde	0.431	0.544	0.899	0.788
Ammonia N mg/l	**0.001**		0.435		0.159			**0.001**	**0.018**	0.780	**0.010**
Ammonia N-NH_3_ mg/l	**0.001**		0.331		0.149			**0.001**	**0.016**	0.705	**0.012**
Ammonia N-NH_4_ mg/l	**0.001**		0.428		0.143			**0.001**	**0.016**	0.732	**0.010**
Nitrates NO_3−_ mg/l	**0.002**		**0.001**	de	**0.007**		cde	**0.001**	0.199	0.518	0.310
Nitrates N mg/l	**0.014**		**0.001**	de	**0.002**		cde	**0.001**	0.242	0.900	0.239
PO_4_^3−^ mg/l	**0.001**		0.427	abcd	0.830		cd	**0.032**	0.317	0.319	0.817
P mg/l	**0.001**		0.642	bcd	0.921		cd	**0.008**	0.403	0.516	0.736

High flows significantly decreased values of pH, TDS, and ammonia concentrations. In the weeks following high flows, ammonia concentrations were significantly lower, and flow was significantly higher compared to the weeks before high flow. Mean values of water temperature, COD, oxygen parameters, CaCO_3_, and nitrates were minimally affected by high flows, while other mean values of variables decreased except for sulphate (although not significantly).

Despite the flood event and high flows, seasonal trends were still expected. Seasonal (spring, summer, autumn, and winter) and monthly differences were computed for the entire dataset and for each year (Table 1). For the monthly differences over 1 year, the effect of a small dataset (only four weekly measurements) must be considered. Nevertheless, significant seasonal and monthly differences in each year were confirmed for water temperature, flow, DO, TDS, COND, CaCO_3_, and sulphates. Nitrates and pH showed seasonal/monthly differences, but only after the flood.

Flows were low in the colder months (December to the end of March). During this period, high values of pH, DO, COND, TDS, CaCO_3_, sulphates, and nitrates were measured. Flow was positively correlated (in all years) with water temperature and negatively correlated with pH, DO, COND, TDS, CaCO_3_, and sulphates (Table 2). Correlation coefficients varied between seasons. For example, a high flow increased water temperature in spring and autumn but decreased it in summer (especially in June). Conversely, DO decreased with a high flow in spring and autumn and increased in summer (in June). TDS values decreased during spring snowmelt in April and again between autumn and winter (September to January), particularly during precipitation events and with snowmelt. Similarly, CaCO_3_ decreased with a high flow in all seasons, most significantly between January and April. Sulphates, which were high in winter, also decreased with high flow in spring. Values of pH decreased with high flow in autumn (October and November), spring, and specifically in June during rainy periods. Nitrates only significantly decreased in autumn in 2019 and 2021, suggesting that concentrations of (our monitored) various compounds decrease with increasing flows in the stream.

**Table 2:** Correlation of the flow (m^3^ s^−1^) with measured variables with regards to seasonality in the mountain stream Javorinka. Significant correlations are in bold. Letters in column ‘Years’ indicate significant (+ positive, − negative) correlation in individual years of monitoring (a, 2017; b, 2018; c, 2019; d, 2020; e, 2021). Numbers in column ‘Months’ indicate significant (+ positive, − negative) correlation in individual months

Flow	All data	Years					Winter	Spring	Summer	Autumn	Months										
*t*	**0.62**	+ a	+ b	+ c	+ d	+ e	0.25	**0.64**	** − 0.41**	**0.43**			+ 3	+ 4		− 6				+ 11	
COD	0.09					+ e	− 0.22	0.21	− 0.03	0.20		− 2				− 6		+ 9	+ 10		
pH	** − 0.39**		− b	− c	− d	− e	− 0.22	** − 0.36**	** − 0.26**	** − 0.54**						− 6			− 10	− 11	
DO	** − 0.59**	− a	− b	− c	− d	− e	− 0.11	** − 0.53**	**0.36**	** − 0.39**			− 3			+ 6					− 12
*p*O_2_	** − 0.15**					− e	**0.26**	− 0.08	0.09	− 0.18	+ 1	+ 2				+ 6					
DO_%_	− 0.11		− b			+ e	**0.41**	− 0.13	0.14	− 0.16	+ 1	+ 2									
COND	** − 0.79**	− a	− b	− c	− d	− e	** − 0.42**	** − 0.70**	** − 0.48**	** − 0.84**	− 1			− 4			− 8	− 9	− 10	− 11	− 12
TDS	** − 0.79**	− a	− b	− c	− d	− e	** − 0.39**	** − 0.72**	** − 0.44**	** − 0.84**	− 1			− 4			− 8	− 9	− 10	− 11	− 12
Cl	− 0.03						− 0.11	0.18	− 0.03	0.10				+ 4							
NaCl	− 0.02						− 0.09	0.17	− 0.03	0.09				+ 4							
CaCO_3_	** − 0.68**	− a	− b	− c	− d	− e	** − 0.43**	** − 0.72**	** − 0.48**	** − 0.43**	− 1	− 2	− 3	− 4			− 8		− 10		
SO_4_^2−^	** − 0.49**	− a	− b	− c	− d	− e	** − 0.32**	** − 0.52**	** − 0.29**	− 0.13	− 1						− 8				
S	** − 0.49**	− a	− b	− c	− d	− e	** − 0.29**	** − 0.57**	** − 0.27**	− 0.11	− 1			− 4			− 8				
Ammonia N	− 0.06						0.13	− 0.11	− 0.01	− 0.14			− 3		− 5						
Ammonia N-NH_3_	− 0.06						0.16	− 0.10	− 0.02	− 0.14			− 3		− 5						
Ammonia N-NH_4_	− 0.06			− c			0.10	− 0.14	− 0.03	− 0.14			− 3		− 5						
Nitrates NO_3−_	** − 0.25**			− c		− e	− 0.19	− 0.10	− 0.05	** − 0.26**											
Nitrates N	** − 0.28**			− c		− e	− 0.23	− 0.15	0.02	** − 0.30**											
PO_4_^3−^ l	0.05		− b	+ c			− 0.02	0.07	− 0.09	− 0.06			+ 3								
P	0.04						0.01	0.07	− 0.08	− 0.08			+ 3								

**Fig. 1:**
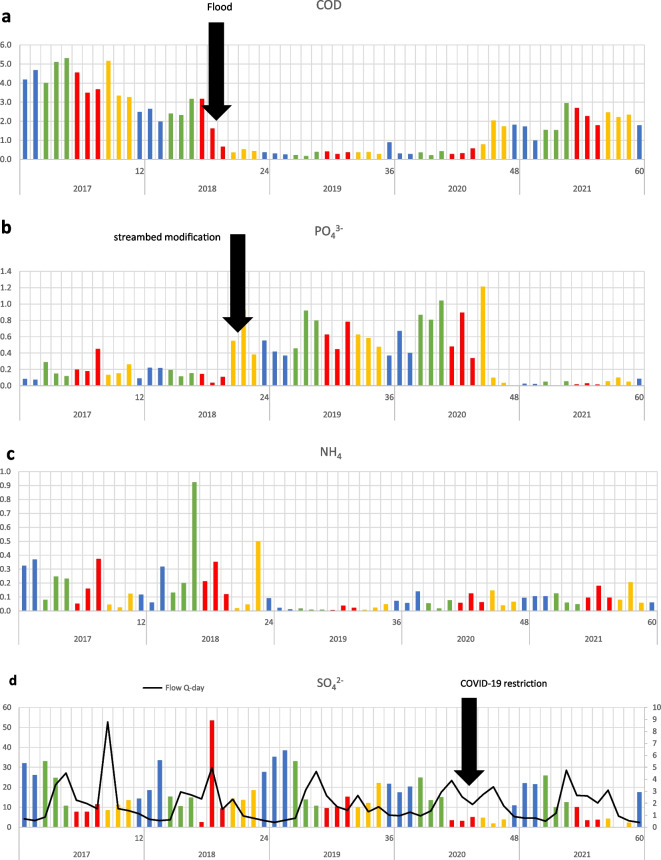
Monthly data trends of **a** chemical oxygen demand (COD in mg/l) influenced by development after flood (18.07.2018), **b** phosphates (PO_4_^3−^ in mg/l) influenced by stream bed erosion after flood; **c** ammonia (NH_4_ in mg/l) influenced by stream bed erosion after flood, and **d** sulphates (left axis SO_4_^2−^ in mg/l; right axis Flow in m^3^/s) influenced by COVID-19 tourism restriction (monthly mean values, in mg/l; green, spring; red, summer; orange, autumn; blue, winter)

**Fig. 2:**
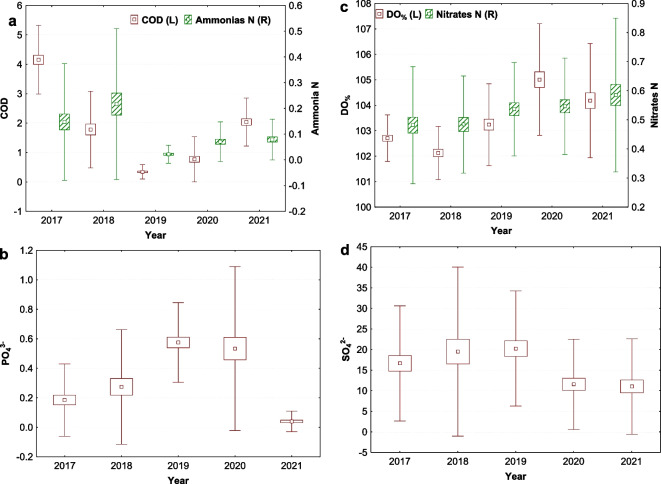
Annual mean values of variables influenced by the flood event and consequent streambed modification. **a** Chemical oxygen demand (COD in mg/l; K-W H (4, 249) = 163.594, *p* = 0.001) and ammonias (N in mg/l; K-W H (4, 249) = 48.911, *p* = 0.001). **b** Phosphates (PO_4_^3−^ in mg/l; K-W H (4, 249) = 110.678, *p* = 0.001). **c** Dissolved oxygen saturation (DO_%_ in %; K-W H (4, 246) = 83.954, *p* = 0.001) and nitrates (N in mg/l; K-W H (4, 249) = 12.478, *p* = 0.014). **d** Sulphates (SO_4_^2−^ in mg/l; K-W H (4, 249) = 23.005, *p* = 0.001) (middle points, means; boxes, + / − standard errors of mean; whiskers, + / − standard deviations of mean)

Surprisingly, chemical oxygen demand (COD), chlorides, ammonias, and phosphates were not significantly correlated with flow and did not vary significantly between seasons or months, but did vary significantly between years. For example, the relationship of phosphates with flow was significantly different between 2018 and 2019 (before and after the flood). In 2018, high flow decreased phosphate concentrations, while in 2019 (after the flood) high flow increased leaching of phosphates into the stream. The phosphates had a high correlation coefficient with COD (− 0.49). Therefore, monthly mean data of COD and phosphates from the dataset were plotted on graphs (Fig. 1a and b). We can see a significant decrease in COD values caused by the flood over the next 2 years (Figs. 1a and 2a). In contrast, phosphates increased over the same period (Figs. 1b and 2b), which could be related to erosion of the stream bed and high flushing of soil along the bank, or streambed modification carried out by the Slovak Water Management Company after the flood. Similarly, ammonia concentrations (Figs. 1c and 2a) were decreased by the flood, especially in 2019. On the other hand, nitrates continually increased year-over-year (*R*^2^ = 0.122), as did values of dissolved oxygen saturation (*R*^2^ = 0.276; Fig. 2c).

**Fig. 3:**
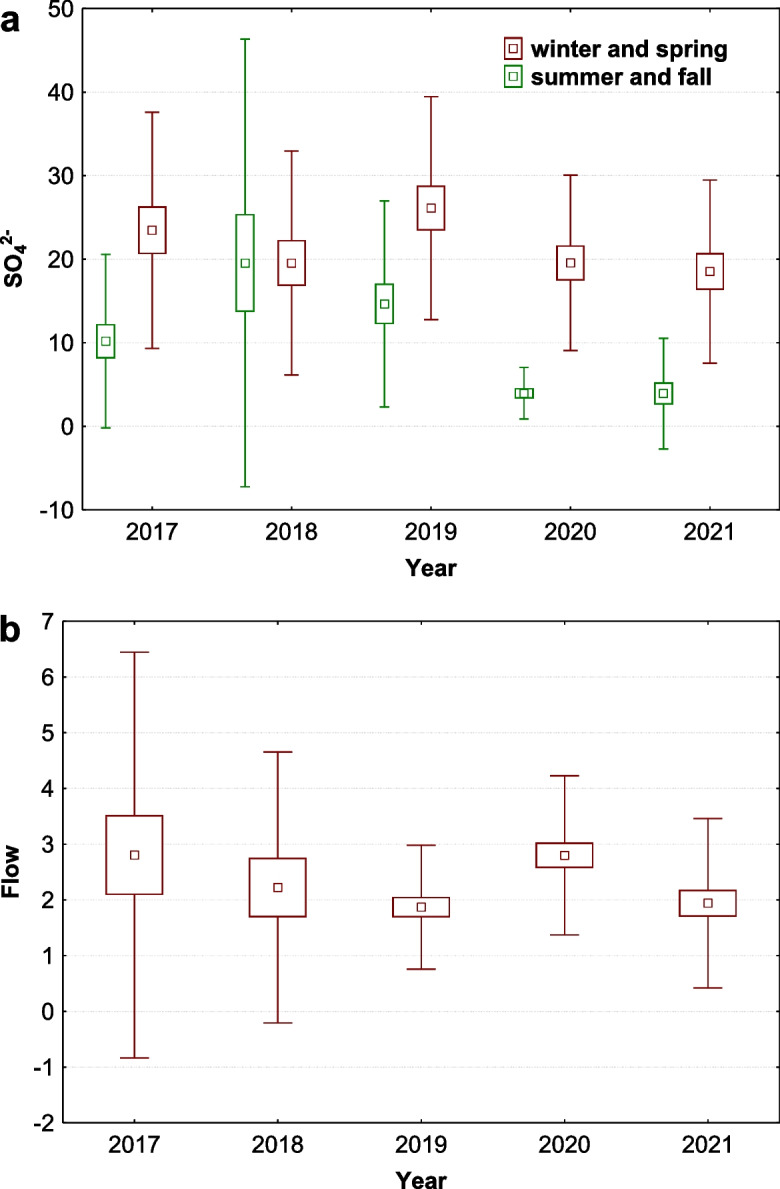
Comparison of summer and autumn data with winter and spring data of **a** annual mean values of sulphates (SO_4_^2−^ in mg/l winter and spring, K-W H (4, 124) = 6.029, *p* = 0.197; summer and autumn, K-W H (4, 125) = 45.339, *p* = 0.001) and **b** means of day flow (flow in m^3^/s: K-W H (4, 166) = 14.407, *p* = 0.006) at site Javorina (middle points, means; boxes, + / − standard errors of mean; whiskers, + / − standard deviations of mean)

Five days after the flood, measurements showed the flow was still five times higher than usual (Fig. 1c), and sulphate concentration increased to 95 mg/l (mean values for year before flood was 7.75 mg/l), the most significant increase of all variables. Despite this, seasonal variations of sulphates continued to follow a consistent trend, confirmed by each year. Higher values were observed in colder months, from November to March, and lower values were observed between April and October, respecting the trend of seasonal flow of the stream. Interestingly, sulphates were significantly low in summer and autumn of 2020 and 2021 (Figs. 1d and 2d). To test these differences, data on sulphates from winter and spring were compared with data from summer and autumn. The result confirmed that sulphate content was low in summer and autumn of 2020 and 2021 (Fig. 3a), although the flow (correlation of flow with sulphates − 0.49) remained unchanged (Fig. 3b).

### Principal component analysis and factors altered physicochemical parameters

**Fig. 4:**
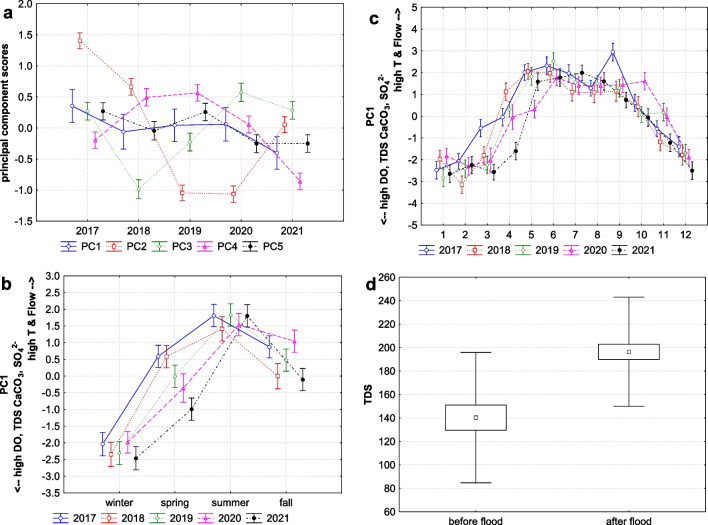
**A** Principal component scores by years; PC1: *F* (4, 241) = 1.072, *p* = 0.371; PC2: *F* (4, 241) = 69.809, *p* = 0.001; PC3: *F* (4, 241) = 16.705, *p* = 0.001; PC4: *F* (4, 241) = 18.825, *p* = 0.001; PC5: *F* (4, 241) = 3.359, *p* = 0.011. **b** Seasonal trend of the first factor (PC1: *F* (3, 242) = 108.59, *p* = 0.001) indicates that higher water flow and temperature (*T*) are associated with lower concentrations of dissolved oxygen (DO), total dissolved solids (TDS), calcium carbonate (CaCO_3_) and sulphates (SO_4_^2−^). **c** Monthly trend of the first factor (PC1: *F* (3, 234) = 73.744, *p* = 0.001) (middle points, LS means; vertical bars denote + / − standard errors). **d** Concentrations of total dissolved solids (TDS in mg/l; K-W H (1, 74) = 15.530, *p* = 0.001) before and after flood in 2018 (middle points, means; boxes, + / − standard errors of mean; whiskers, + / − standard deviations of mean)

Principal component analysis (PCA) was performed to better estimate the mutual relationships between the measured values. The resulting components (Table 3, Fig. 4a) could represent physicochemical parameters present in the stream that could be influenced by seasons or flooding. The first factor (27.03% of the total variance) shows a strong positive association with flow and temperature, and a strong negative association with dissolved oxygen (DO), total dissolved solids (TDS), and calcium carbonate (CaCO_3_), and also partly with sulphates (SO_4_^2−^) and nitrates (NO_3_). This suggests that higher water flow and temperature are associated with lower dissolved oxygen, total dissolved solids, calcium carbonate levels, and partly with sulphates and nitrates. This factor is significantly influenced by seasonal and monthly changes, particularly in spring or winter (Fig. 4a, b, and c). This seasonal and monthly cycle is very similar in each year (Fig. 4b), but in the spring before (2017–2018), and after the flood (2019–2021), slight differences in direction of factors were observed (*F* (4, 55) = 1.8606, *p* = 0.130). Differences for spring temperature and flows between years were tested, but they were not significant (K-W H test for water temperature (4, *N* = 63) = 3.745 *p* = 0.442; K-W H test for Flow (4, *N* = 63) = 2.048, *p* = 0.727). However, when other variables related to this factor were tested, TDS concentrations significantly and gradually increased from 2017. Comparing these concentrations in spring before and after the flood, TDS concentrations were significantly higher after the flood (KW-H (1, 74) = 15.530, *p* = 0.001, Fig. 4d). Thus, spring flows tend to flush more total dissolved solids into the stream after the flood.

**Table 3:** Resulting principal component (PCA) physicochemical properties of mountain stream Javorinka in the Slovakian Tatra Mountains. Factor coordinates greater than 0.4 or less than − 0.4 in each PC columns are in bold

Variables	PC1	PC2	PC3	PC4	PC5
Flow day mean m^3^/s	**0.613**	0.159	**0.418**	0.251	− 0.092
*t* °C	**0.838**	− 0.220	− 0.287	− 0.206	0.030
COD mg/l	0.153	**0.785**	0.216	− 0.194	− 0.006
pH	− 0.306	− 0.042	** − 0.594**	− 0.324	− 0.157
DO mg/l	** − 0.829**	0.159	0.346	0.160	− 0.095
DO_%_	− 0.196	** − 0.433**	**0.452**	− 0.189	** − 0.470**
TDS mg/l	** − 0.814**	− 0.208	− 0.142	− 0.086	0.145
NaCl mg/l	0.039	0.287	− 0.325	**0.621**	− 0.295
CaCO_3_ mg/l	** − 0.741**	0.151	− 0.046	− 0.131	0.299
SO_4_^2−^ mg/l	** − 0.488**	0.164	− 0.109	**0.459**	0.092
Ammonia N-NH_4_ mg/l	0.032	**0.458**	** − 0.411**	− 0.056	** − 0.442**
Nitrates NO_3−_ mg/l	− 0.357	− 0.212	0.038	− 0.072	** − 0.603**
PO_4_^3−^ mg/l	0.120	** − 0.648**	− 0.157	**0.455**	0.062
Eigenvalue	3.514	1.754	1.315	1.146	1.034
Total variance %	27.030	13.496	10.118	8.814	7.950

**Fig. 5:**
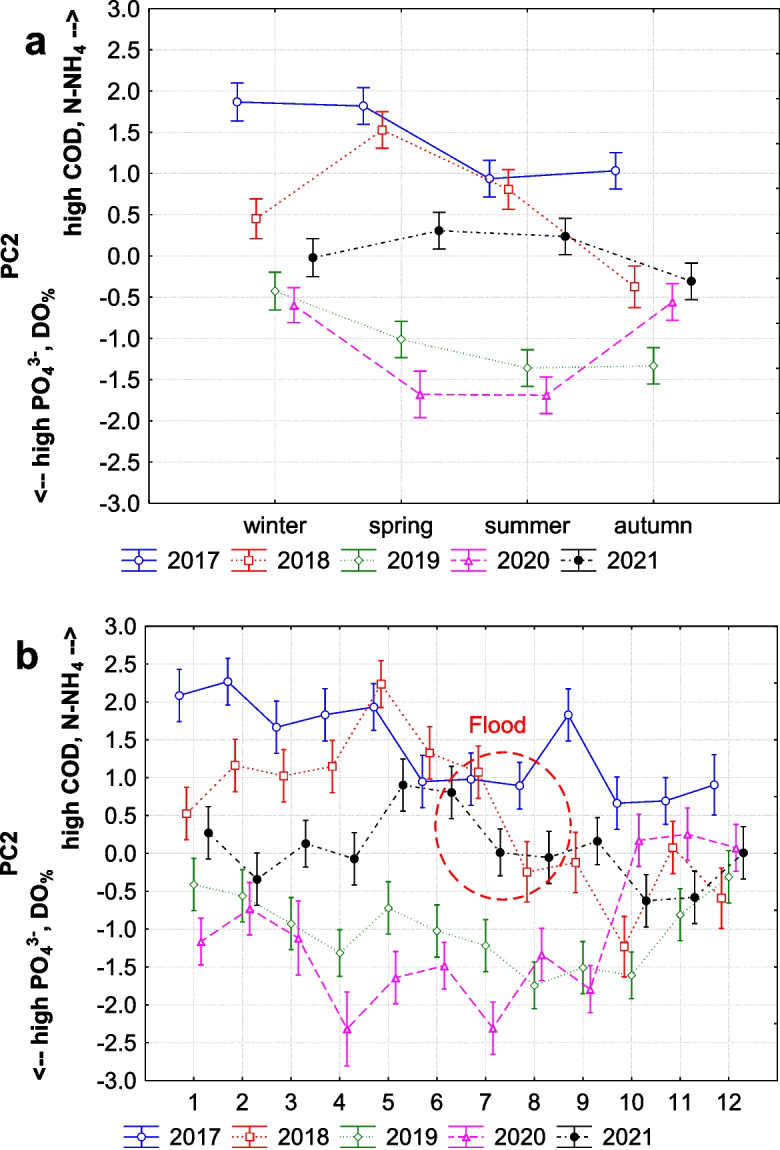
The second factor (PC2) explaining trends of chemical oxygen demand (COD) with ammonia (N-NH_4_) which were substituted by phosphates (PO_4_^3−^) after flood (in July 2018) in the stream. **a** Seasonal trend (*F* (3, 242) = 3.918, *p* = 0.009; interaction with years: *F* (12, 226) = 5.079, *p* = 0.001) and **b** monthly trend (*F* (11, 234) = 1.726, *p* = 0.069; interaction with years: *F* (44, 186) = 4.034, *p* = 0.001) (middle points, LS means; vertical bars denote + / − standard errors)

The second factor (13.50% of the total variance) has a strong positive loading for chemical oxygen demand (COD) and ammonia (N-NH_4_), and a strong negative loading for phosphate (PO_4_^3−^) and dissolved oxygen saturation (DO_%_). This indicates that higher levels of chemical oxygen demand and ammonia are associated with lower levels of phosphate and oxygen in water. This factor exhibits significant yearly (Fig. 4a) and seasonal variation (Fig. 5a and b), suggesting that changes in these organic compounds are more pronounced over longer time scales. These compounds were specifically changed by the flood (Fig. 5b), indicating that organic compounds were removed or flushed out, and the subsequent erosion of the stream bed was so significant that organic compounds with ammonia were substituted by phosphates in the stream.

**Fig. 6:**
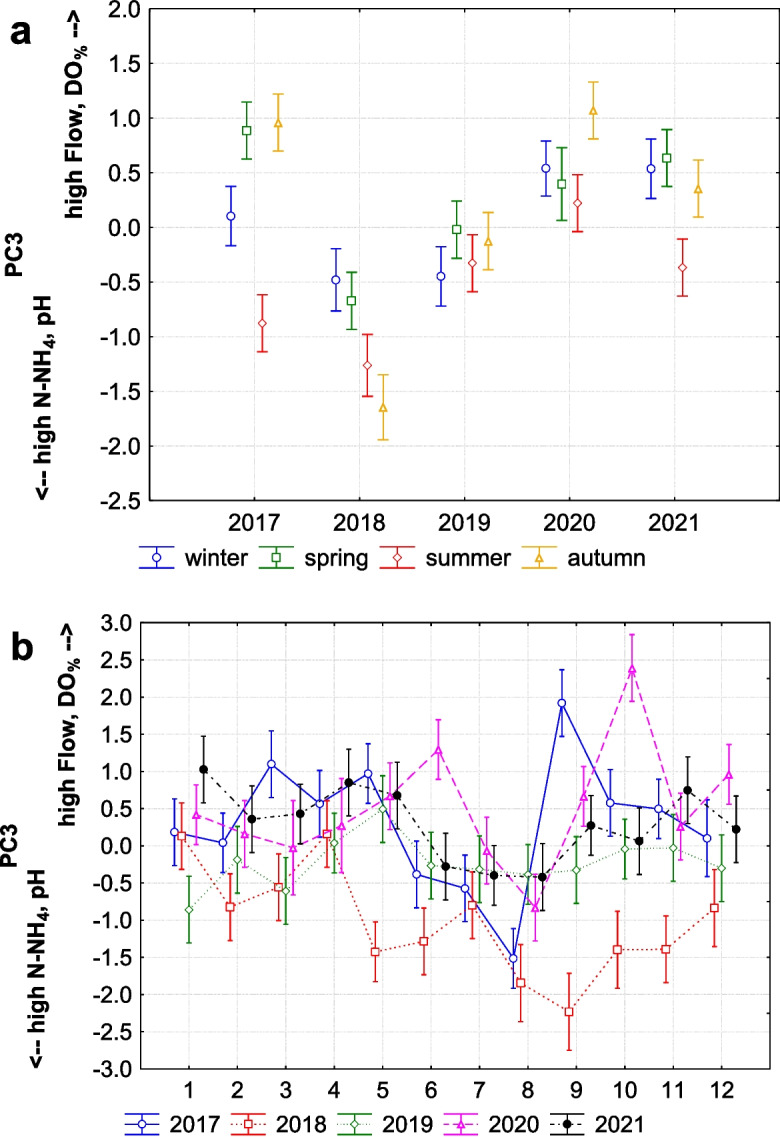
The third factor (PC3) explaining how flow in the stream changing the levels of dissolved oxygen saturation (DO_%_) and pH together with concentration of ammonia (N-NH_4_), **a** seasonal trend (*F* (3, 242) = 5.889, *p* = 0.001; interaction with years: *F* (12, 226) = 2.650, *p* = 0.002); **b** monthly trend (*F* (11, 234) = 2.369, *p* = 0.009; interaction with years: *F* (44, 186) = 1.922, *p* = 0.001) (middle points, LS means; vertical bars denote + / − standard errors)

The third factor (10.12% of the total variance) has a strong positive loading for flow and dissolved oxygen saturation (DO_%_), and a strong negative loading for pH and ammonia (N-NH_4_). This suggests that the high flow increased dissolved oxygen saturation and decreased pH levels with concentration of ammonia, or vice versa. This factor demonstrates significant yearly (Fig. 4a), seasonal, and monthly variation (Fig. 6a and b), indicating consistent fluctuations in these parameters over all temporal scales. Additionally, this factor may explain contrast between dry and wet periods in catchment, particularly between wet spring or autumn and dry summer. This contrast is especially visible in 2017 before flood (Fig. 6a) and again in 2021 (partly also for summer vs. autumn in 2020) 2 years after the flood event.

**Fig. 7:**
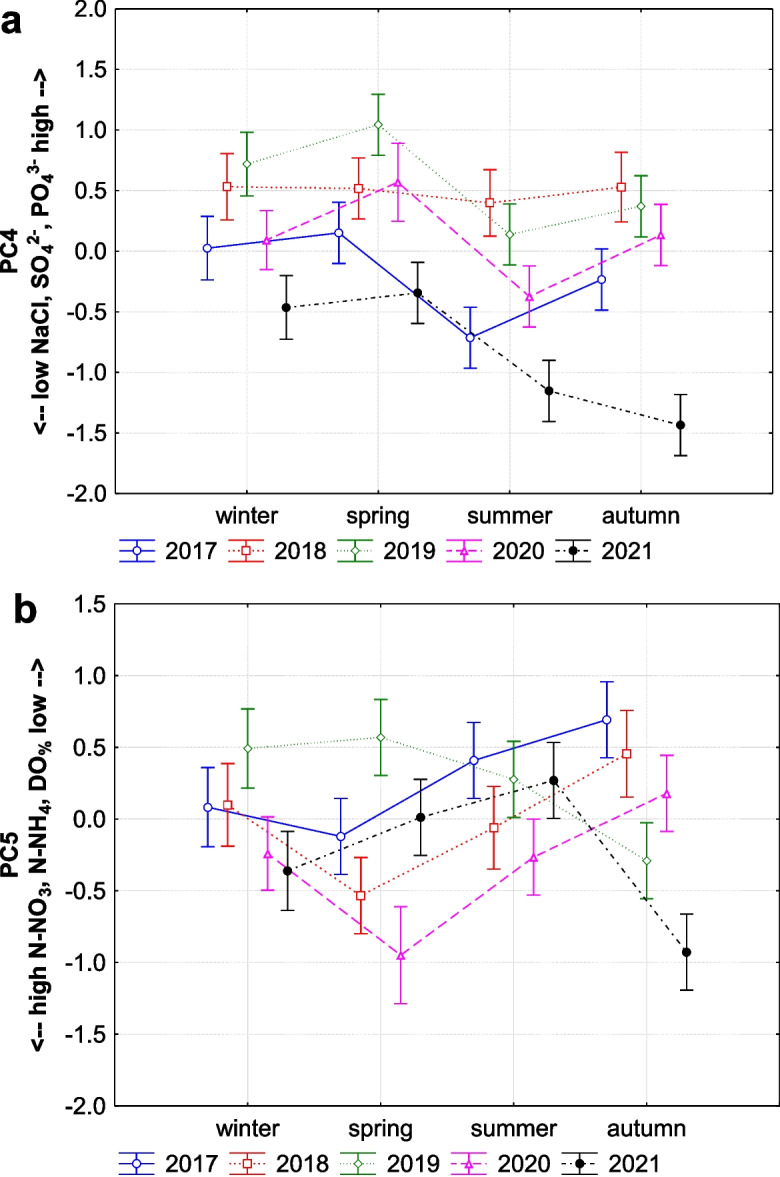
**A** The seasonal trend of the fourth factor (PC4) explaining strong positive association of chlorides (NaCl), sulphates (SO_4_^2−^), and phosphates (PO_4_^3−^) in the stream (*F* (3, 242) = 6.208, *p* = 0.001; interaction with years: *F* (12, 226) = 0.808, *p* = 0.642). **b** The seasonal trend of the fifth factor (PC5) explaining nitrification processes in the stream due to strong association of nitrates (N-NO_3_), ammonia (N-NH_4_), and dissolved oxygen saturation (DO_%_) (in 2017: *F* (3, 47) = 1.742, *p* = 0.171; in 2018: *F* (3, 41) = 1.265, *p* = 0.299; in 2019: *F* (3, 47) = 3.750, *p* = 0.017; in 2020: *F* (3, 44) = 3.242, *p* = 0.031; in 2021: *F* (3, 47) = 3.617, *p* = 0.038) (middle points, LS means; vertical bars denote + / − standard errors)

The fourth factor (8.81% of the total variance) shows a strong positive association with chlorides (NaCl), sulphates (SO_4_^2−^), and phosphates (PO_4_^3−^), indicating that higher levels of these parameters tend to occur together. This factor shows significant variation across all temporal scales (Figs. 4a and 7a), with a consistent pattern of seasonal trends in individual years, except in 2018 (the year of the flood). A strong seasonal contrast was observed between spring and summer (Fig. 7a), also except in 2018. In 2019, after flood, this factor achieved highest positive loading, indicating that concentrations of chlorides, sulphates, and phosphates were higher after the flood than before in 2017 or after 2 years in 2021.

The fifth factor (7.95% of the total variance) shows a strong negative association with nitrates (N-NO_3_), ammonia (N-NH_4_), and dissolved oxygen saturation (DO_%_), indicating that higher levels of nitrates are strongly associated with higher ammonia and dissolved oxygen saturation in water. This interesting interplay between these variables may explain nitrification processes in our stream, because ammonia and oxygen saturation are critical predictors for nitrification and enhance the activity of nitrifying bacteria, facilitating the conversion of ammonia to nitrate. When these conditions are favourable (i.e. high ammonia and high DO_%_), nitrification rates increase, leading to higher nitrate concentrations. The negative loadings might reflect periods or conditions where ammonia is being rapidly converted to nitrate. This factor exhibits significant seasonal and monthly variations only in the years following the flood (2019–2021; Fig. 7b). Especially the highest increases were observed in autumn 2019, spring 2020, and autumn 2021. This suggests that these nutrients (nitrates and ammonia) can be more stable through seasons, but also variable by years, especially after flood events, as a result of ecological recovery in the stream.

## Discussion

The Tatra Mountains are increasingly affected by rising air temperatures. Pribullová et al. (2013) reported a significant linear increase in annual air temperature of 0.21–0.30 °C per decade from 1961 to 2007, with notable increases in maximum and minimum daily air temperatures during summer and winter months (July, August/December, January). Studies by Melo et al. (2009) and Łupikasza and Szypuła (2019) corroborate this upward trend in vertical climatic zones over recent decades. Furthermore, Bičárová and Holko (2013) noted a significant rise in precipitation at high elevations and an increase in the number of days with precipitation. Górnik et al. (2017) highlighted a significant increase in the number of days with potentially dangerous precipitation (40 to 60 mm per day), and Földes et al. (2020) predicted that short-term rainfall intensities would become more extreme in future. These climate observations and future scenarios suggest a continuing influence on the hydrological parameters of alpine/mountain streams in the Tatra Mountains.

Long-term observations of daily river discharges associated with flood events have shown substantial differences across individual catchments (Pociask-Karteczka et al., 2010; Bičárová & Holko, 2013). The windward side of the Tatra Mountains, due to the barrier effect for air masses coming from northwest and west (see Niedźwiedź et al., 2015), is particularly vulnerable to extreme flood risks (Bičárová & Holko, 2013; Górnik et al., 2017; Holko & Pociask-Karteczka, 2019). Ruiz-Villanueva et al. (2016) analysed the seasonality of flood magnitudes over the past 60 years in the northern foreland of the Tatra Mountains, finding a decreasing trend in snowmelt floods in winter and increasing trends in flood magnitudes in spring and autumn. They expect that while floods might become less frequent, they will likely be more extreme in future periods.

These expectations materialised in July 2018, when a significant flash-flood (217.9 mm, SHMÚ, 2018) impacted the mountain stream monitored in this study. This event provided a unique opportunity to evaluate the flood’s impact on processes within alpine/mountain stream ecosystems. Previous studies by Hrivnáková (2019) and Hrivnáková et al. (2020) evaluated the shallow mountain lake Kolové pleso (1565 m a.s.l., 2 ha, max. depth 1.1 m), within the watershed of our investigated stream and observed that the flood washed out deposited sediments and sharply decreased the organic matter content in the lake. Similar effects were observed in the Javorinka stream.

Flash floods are highly variable hydrological processes and are generally associated with a large sediment discharge over short period (Ballesteros-Cánovas et al., 2015). Sediments significantly influence the saturation of water by organic and inorganic compounds (Bucher et al., 2017; Hamid et al., 2020). Rączkowska et al. (2024) focused on the geomorphological impacts of the same July 2018 flood in the Polish Tatra Mountains (catchment area of the Sucha Woda stream) and confirmed that the upper part of the stream channel was cleared of fine material (such as fine sand and small gravel).

Alpine/mountain catchments, along with their lakes, act as sinks or sources of organic and inorganic matter to downstream waters throughout the seasons (Ejarque et al., 2018; Goodman et al., 2011; Vigiak et al., 2019). This process is complex and closely related to physical (erosion) and chemical (weathering) processes in the mountains, primarily through water–rock and water-organic matter interactions linked to solubility of minerals present in the entire catchment (Bucher et al., 2017). Our study found significant seasonal differences in total dissolved solids (TDS) values each year. This is a common factor (PC1), related to both temperature and flow in the stream. Alpine/mountain streams tend to be richer in TDS during the winter months due to the high contribution of groundwater (Donnini et al., 2016), as this is when flows are reduced by freezing water in structures of catchments (Siwek & Żelazny, 2019). Interestingly, this factor changed in the spring following the flood, and we observed that spring flows becoming richer in TDS, likely due to the eroded river bank and enhanced chemical weathering (Litaor, 2022; Zobrist, 2010).

On the other hand, values of chemical oxygen demand (COD) and ammonia compounds were significantly low after the flood during spring and summer (PC2). This clearly indicates that the flash-flood flushed a significant amount of organic as well as inorganic matter from the stream. Flash floods are highly dynamic events that rapidly increase water flow (Ballesteros-Cánovas et al., 2015), causing substantial erosion of stream banks and surrounding soils (Rączkowska et al., 2024). During such events, the increased kinetic energy of the water mobilises and transports large quantities of particulate and dissolved organic matter from the soil and stream bed into the water column. This process is often accompanied by the physical breakdown of soil aggregates, releasing bound organic and inorganic substances. In our study, 5 days after the flood, when the flow was still five times higher than usual, we measured significantly high concentrations of sulphates in the stream. This sudden increase in sulphates can be attributed to the rapid erosion and flushing of topsoil layers, where sulphur compounds are more abundant. Soil investigations in the Tatra Mountains (Kopáček et al., 2006) have shown that sulphur is primarily concentrated in the topsoil horizons. When these topsoil layers are eroded, sulphur compounds are released into the stream. This was followed by increased concentrations of phosphates for almost 2 years, as also visible in seasonal trends of PC2. Phosphorus, like sulphur, is also more abundant in the topsoil layers, and its mobility is influenced by soil erosion processes. The flash flood significantly depleted the entire catchment of organic matter, including phosphorus. In addition, undeveloped till soils in scree areas represent an important source of mobile phosphorus forms for waters in alpine catchments (Kaňa et al., 2023). As the floodwaters receded, the phosphates released from eroded soils and decomposed organic matter remained in the stream, leading to prolonged elevated PO4 concentrations.

Heavy rain-induced floods in summer months often result in biomass decline (Robinson & Uehlinger, 2008; Uehlinger et al., 2003). In addition, this type of flood may be considered a natural disturbance factor that maintains oligotrophic conditions in alpine/mountain streams. Although we did not directly measure biomass, we strongly suspect that this was the case. After the flood, we observed increased oxygen saturation in the stream, which may indicate changes in the stream bed and improved aeration (by wind, waves, rapids, waterfalls, or groundwater discharge). Alternatively, this could be caused by low consumption of oxygen by biota in the stream, particularly periphyton, due to supressed processes of ammonification (due to low resources to decompose) and the reduced microbial activity of ammonia-oxidising bacteria and archaea (Auguet et al., 2011; Schleper & Nicol, 2010), which typically reduce oxygen in water through aerobic decomposition on ammonia. After the flood, decreased chemical oxygen demand and ammonia concentration can support this idea. Despite the reduction of ammonia due to the flood, we observed an interesting factor, interplay between nitrates, ammonia, and oxygen saturation (PC5), which may explain nitrification processes in our stream, because ammonia and oxygen saturation are critical predictors for nitrification and enhance the activity of nitrifying bacteria, facilitating the conversion of ammonia to nitrate. Consequent increasing ammonia concentrations, suppressed by the flood, in the stream after the 2 years following the flood likely reflect a high regrowth of plants/algae, high productivity of oxygen (due to photosynthesis, respiration), and a high accumulation of organic matter available for decomposition. This mutual interaction of increasing nitrates, ammonia, and oxygen suggests the stream ecology recovery from the flood and the resilience of alpine and mountain stream ecosystems in the face of extreme hydrological events. Our findings support the hypothesis that floods are essential for maintaining the oligotrophic profile of alpine/mountain streams. The decrease in organic matter and changes in nutrient levels post-flood indicate that such events flush out excess nutrients and sediments, thus preserving the stream’s low-nutrient status. These results align with previous studies (e.g. Bucher et al., 2017; Hamid et al., 2020) that highlight the role of floods in nutrient cycling and stream health.

Alpine/mountain streams serve as information pathways, offering insights into the environmental processes occurring in their ecosystems. These insights include both natural processes and anthropogenic influences. For example, Siwek and Biernacki (2016) observed high loads of biogenic compounds during the substantial influx of tourists to the Tatra Mountains from May to September. In our study, we noted annual seasonal variability in sulphate levels, with higher values during the colder months each year. However, in 2020 and 2021, we recorded significantly lower sulphate values in the summer and autumn compared to the same period in 2017–2019. This change could likely be due to the travel and tourism restrictions imposed during the COVID-19 pandemic or due to decreased emissions from transboundary sources. Similar effects were observed in the Alps, where Rogora et al. (2022) reported significantly lower concentrations and deposition of sulphates and nitrates during the pandemic compared to the previous decade. While our sulphate data align with these findings, the increase in nitrate levels in our study suggests other influencing factors, as long-term trends indicate decreasing nitrate levels in the Tatra Mountains (Kopáček et al., 2019). In our study area, a key factor contributing to increased nitrate levels could be deforestation, particularly after wind events and bark beetle infestations, as documented by Želazný et al. (2017). They found that nitrate concentrations in stream water increased significantly, especially outside the vegetation season. Similar forest degradation has been observed in our catchment (Mezei et al., 2017; Solár & Stražovec, 2021).

Paleohydrological studies in the Tatra mountains (Ballesteros-Cánovas et al., 2015, 2016; Zielonka et al., 2008) confirm that flash floods are a recurring natural phenomenon, expected to increase in frequency and severity due to ongoing and future climate change. Gorczyca et al. (2014) inferred that rainfall with enough energy to cause flood events occurs every 6–9 years at middle elevations in the Tatra Mountains. Therefore, we can expect another significant flood in the near future. Future changes in this ecosystem will reveal whether the observed parameters will continue to support the regular, repeating patterns and mutual interactions that maintain the oligotrophic profile of alpine and mountain streams.

## Conclusion

Every week monitoring of water stream conditions, based on physicochemical properties, aims to determine the extent of seasonal changes, define repeating patterns (cycles) and mutual interactions, and observe the development of conditions following extreme or accidental events that can significantly affect dynamic trends. Over 5 years of monitoring, we recorded well-known seasonal trends of physicochemical parameters correlating with stream flow and temperature. The July 2018 flash flood not only significantly altered the stream morphology, but also significantly affected the trajectories of organic and inorganic compounds. In particular, our results showed suppressed levels of chemical oxygen demand and ammonia, while phosphate concentrations increased over the following 2 years. We believe that the flood probably reduced biological activity, especially microbial, in the stream and the wider catchment area. These changes have implications for the entire stream ecology, as they influence habitat structure and nutrient dynamics. However, from a long-term perspective, these events support the oligotrophic profile of alpine and mountain streams.

## Data Availability

The datasets analysed during the current study are available from the corresponding author on reasonable request.
